# Complex vascular anomalies and tissue overgrowth of limbs associated with increased skin temperature and peripheral venous dilatation: parks weber syndrome or PROS?

**DOI:** 10.1186/s41065-021-00217-6

**Published:** 2022-01-04

**Authors:** Li Xin Su, Yi Sun, Zhenfeng Wang, Deming Wang, Xitao Yang, Lianzhou Zheng, Mingzhe Wen, Xindong Fan, Ren Cai

**Affiliations:** Department of Interventional Therapy, Multidisciplinary Team of Vascular Anomalies, Shanghai Ninth People’s Hospital, Shanghai Jiao Tong University, Shanghai, People’s Republic of China

**Keywords:** *PIK3CA*-related overgrowth spectrum, Parks weber syndrome, PROS, *PIK3CA*, Vascular malformation

## Abstract

*PIK3CA*-related overgrowth spectrum (PROS) is a series of congenital, sporadic disorders that are associated with segmental overgrowth phenotypes and postzygotic, somatic gene mutations in the PIK3CA-ATK-mTOR pathway. The variability and overlapping phenotypes between PROS and other complex vascular malformations make the differential diagnosis confusing and challenging. PROS should be considered for the differential diagnosis with other complex vascular malformations and syndromes with a tissue overgrowth phenotype, such as Parkes-Weber syndrome (PWS).

Herein, we diagnosed one unique clinically challenging case manifested as capillary malformation (CM), limb overgrowth, as well as increased skin temperature and peripheral venous dilatation of lower limb that indicated a potential fast-flow lesion. The patient was initially diagnosed with PWS. Contrary to the previous diagnosis, based on further MR imaging and digital subtraction angiography (DSA), which ruled out the existence of AVMs and AVFs, and molecular analysis with targeted next-generation sequencing (NGS) revealing a somatic PIK3CA mutation, we ultimately diagnosed that the patient had a unique form of PROS simulating PWS phenotypes. We suggest that it is important to propose the differential diagnosis of PWS and PROS, two diseases that share a common overgrowth phenotype. We recommended radiological diagnosis such as MRI, CT and DSA as well as further molecular diagnosis to provide more information for the assessment of vascular lesions and to further guide clinical treatment strategies.

## Background

*PIK3CA*-related overgrowth spectrum (PROS), a series of congenital, sporadic disorders that are associated with segmental overgrowth phenotype and postzygotic, somatic gene mutations in the PIK3CA-ATK-mTOR pathway, was defined to encompass multiple, heterogeneous phenotypes at a National Institute of Health (NIH) workshop in 2013 [1–3]. The hyperactivation of PI3K-ATK-mTOR signaling results in various vascular anomalies and abnormal tissue growth [4, 5]. At present, disorders identified as belonging to the PROS include the following: Klippel-Trenaunay syndrome (KTS) [6]; CLOVES (Congenital Lipomatous Overgrowth, Vascular malformations, Epidermal nevi, Scoliosis/Skeletal and Spinal) syndrome [7]; DMEG (congenital lipomatous overgrowth; dysplastic megalencephaly) [8]; FAO (fbroadipose hyperplasia or overgrowth) [9]; FIL (fibroadipose infiltrating lipomatosis/facial infiltrative lipomatosis) [10]; HHML (hemihyperplasia multiple lipomatosis) [11]; and MCAP/M-CM (Megalencephaly Capillary Malformation-Polymicrogyria Syndrome) [12]. The variability and overlapping phenotypes between PROS and other complex vascular malformations make the differential diagnosis confusing and challenging [4, 13]. For example, Parkes-Weber syndrome (PWS), another type of complex vascular malformation characterized by capillary malformation and segmental limb overgrowth, strongly overlaps with KTS, diffused capillary malformation with overgrowth (DCMO) and CLOVES syndrome [14, 15]. The identification of arteriovenous malformations (AVMs) or arteriovenous fistulas (AVFs) in PWS patients contributes to the differential diagnosis of KTS and CLOVES syndrome [16]. Moreover, *RASA1* mutations have been shown to underlie PWS, and genetic study can be essential for differential diagnosis with other vascular overgrowth entities [17].

In this study, we diagnosed a unique PROS characterized by very subtle phenotypes masquerading as PWS. The patient was initially considered to have PWS because of the bilaterally segmentally distributed capillary malformation (CM) on both the lower and upper limbs, chest and back, the hypertrophic lower limbs associated with longitudinal growth, and the increased skin temperature and peripheral venous dilatation of his lower limbs, suggesting a potential fast-flow lesion. Contrary to the previous diagnosis, based on further MR imaging and DSA ruling out the existence of AVMs and AVFs, and molecular analysis with targeted next-generation sequencing (NGS) revealing a somatic *PIK3CA* mutation, we ultimately diagnosed that the patient had a unique form of PROS mimicking PWS phenotypes. Our case contributes to delineating novel clinical features (increased skin temperature and peripheral venous dilatation) that could be identified in association with PROS.

We suggest that in some cases, the diagnosis based on clinical symptoms and signs is restricted. It is important to propose the differential diagnosis of PWS and PROS, two diseases that share a common overgrowth phenotype. We recommended radiological diagnosis such as MRI, CT and DSA as well as further molecular diagnosis to provide more information for the assessment of vascular lesions and to further guide clinical treatment strategies.

## Case presentation

**Fig. 1:**
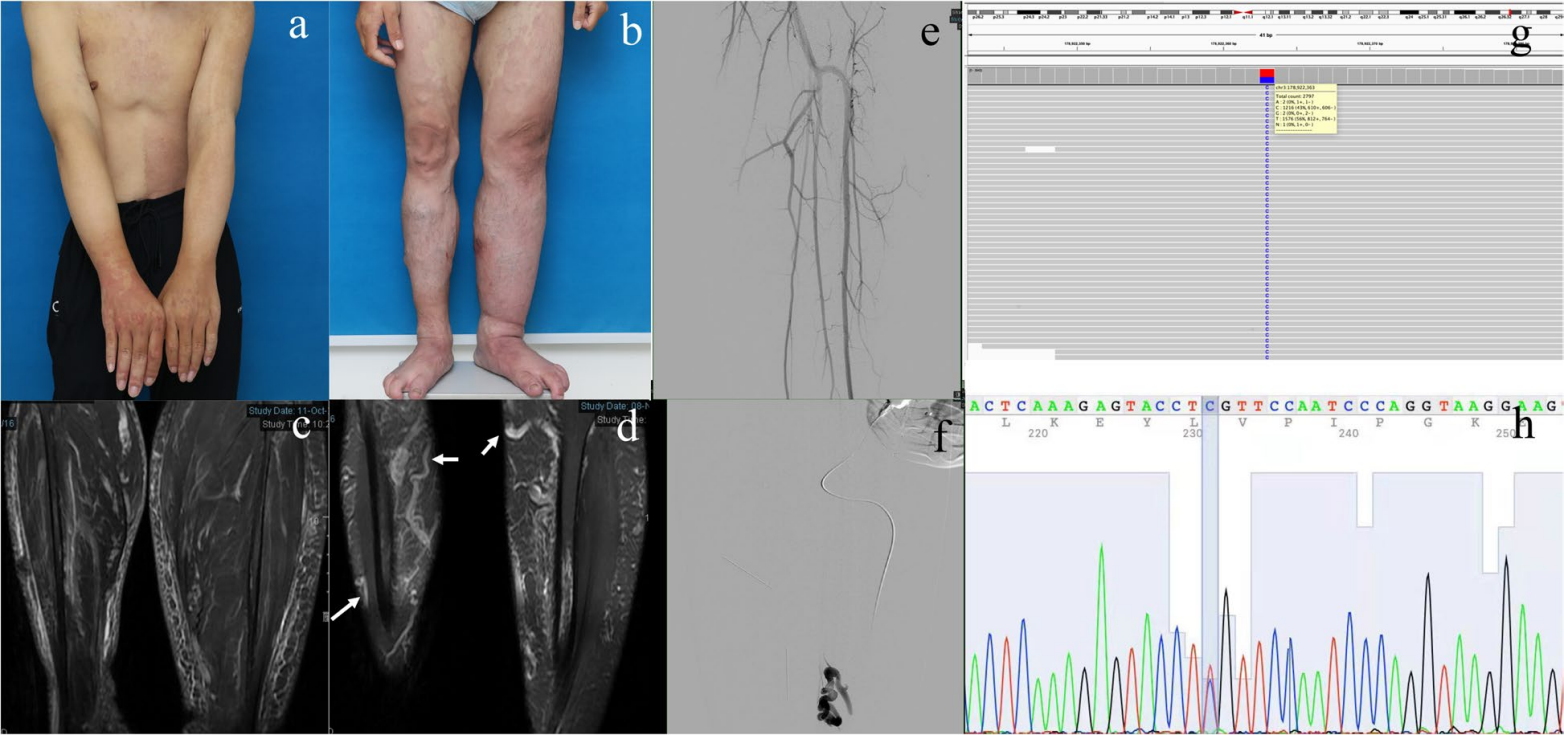
Clinical manifestation of the patient. **a**: Extensive, bilaterally and segmentally distributed capillary malformation on both the lower and upper limbs, chest and back. **b**: Bilateral hypertrophy associated with longitudinal growth, peripheral venous dilatation and increased skin temperature of the lower extremities. **c-d**: MR imaging showed diffuse tissue hypertrophy (1c) and multiple venous dilation (1d, arrows) of soft tissue and muscle of bilateral lower limbs. **e**: Intraarterial digital subtraction angiography (IADSA) showed no obvious AVM/AVF. **f**: Intravenous DSA (IVDSA) showed venous dilation of the soft tissue and muscle. **g**: IGV visualization of NGS data for the tissue sample with somatic mutation in *PIK3CA* (p.Cys378Arg; c.1132 T > C). **h**: Sanger sequence of somatic mutation in *PIK3CA* (p.Cys378Arg; c.1132 T > C)

A 23-year-old male patient visited our vascular anomaly clinic and presented with extensive, bilaterally and segmentally distributed CM on both the lower and upper limbs, chest and back. Bilateral hypertrophy associated with longitudinal growth of the lower extremities presented since birth and became progressively significant with age. Further physical examination revealed peripheral venous dilatation of the lesions on both sides of his lower extremities, and the skin temperature at the lesion was significantly higher (2 °C) than that in the adjacent normal skin (Fig. 1a, b). No obvious neurological abnormalities or dysmorphic features were found during physical examination. According to the patient, family history was unremarkable. Based on clinical symptoms and signs, we suspected that the venous dilatation and increased skin temperature in both lower extremities were secondary manifestations of the epherent hyperflow from the potential high-flow lesions, and diagnosed the patient as Parkes-Weber syndrome. Considering that the potential fast-flow lesions can be damaging, we performed MR imaging to promote further diagnosis and to assess the extent of soft tissue and muscle involvement. MR imaging showed diffuse tissue hypertrophy and multiple venous dilations of soft tissue and muscle of bilateral lower limbs. No obvious arteriovenous fistula were identified on MR imaging (Fig. 1c, d). Intraarterial digital subtraction angiography (IADSA) showed no obvious AVM/AVF (Fig. 1e). Intravenous DSA (IVDSA) showed venous dilation of the soft tissue and muscle (Fig. 1f).

Minimally invasive biopsies were performed under local anesthesia for further molecular diagnosis. Tissue specimens of the CM lesion and peripheral blood specimens were collected and preserved for DNA extraction and NGS. We designed the targeted NGS gene panel based on the ISSVA classification [18].

The genetic results revealed a somatic mutation in *PIK3CA* (p.Cys378Arg; c.1132 T > C). Integrative Genomics Viewer (IGV) visualization of NGS data for the tissue sample with somatic mutation in *PIK3CA* (p.Cys378Arg; c.1132 T > C) is present in Fig. 1g. Sanger sequence of NGS analysis showed a mutation frequency of 43.63% in *PIK3CA* in the patient’s tissue sample (Fig. 1h). Based on the physical examination showing overgrowth and increased skin temperature of lower limbs, the elimination of AVM or AVF by MRI and DSA, and molecular analysis that revealed somatic *PIK3CA* mutation, the patient was eventually diagnosed to have a unique form of PROS characterized by atypical phenotypes strongly overlapping Parkes-Weber syndrome.

## Discussion

Vascular malformations, according to literature, are considered to result from abnormal signaling processes during embryonic development, leading to varying degrees of differentiation vascular plexus cells [19]. In the last two decades, causative gene mutations of a variety of vascular anomalies have been identified, and the origin of the pathophysiological pathways of most malformation types has begun to be elucidated [20]. The PI3K signaling pathway exerts a enormous function on regulating cell growth, proliferation, differentiation and angiogenesis. Somatic activating gene mutations in the PIK3CA/AKT/mTOR signaling pathway result in various vascular malformations and heterogeneous clinical manifestations associated with tissue overgrowth^**1–3**^. According to the classification of vascular anomalies proposed by the International Society for the Study of Vascular Anomalies (ISSVA) [18], several vascular malformations are known to characterized by bone, muscle, and soft tissue overgrowth, with or without nervous system involvement, including Klippel-Trenaunay syndrome (KTS; capillary malformation, venous malformation and lymphatic malformation, and limb overgrowth involving soft tissue hypertrophy and bony abnormalities); megalencephaly capillary malformation (MCAP; vascular abnormalities, hemihyperplasia and brain malformations); CLOVES syndrome (congenital lipomatous overgrowth, vascular malformations, epidermal nevus, spinal/skeletal anomalies/scoliosis syndrome); CLAPO syndrome (capillary malformation of lower lip; lymphatic malformation in the head and neck and partial/generalized overgrowth). With the recognition of gene mutations in the PIK3CA-ATK-mTOR pathway in these complex vascular and overgrowth disorders, the specific term PIK3CA-related overgrowth spectrum (PROS) was proposed to describe these disorders with heterogeneous but overlapping phenotypic features.

Identification of overlapping characteristics including vascular anomalies and overgrowth in syndromes with other specific genetic mutations can be challenging, and PROS can be misdiagnosed. Proteus syndrome is a complex systematic disorder with an *AKT1*-activating mutation that comprise with asymmetric tissue overgrowth involving almost the entire body and skin manifestations such as cerebriform connective tissue nevi and epidermal nevi [21]. Parkes-Weber syndrome is congenital combined vascular anomalies consisting of CM, AVM and overgrowth of extremities [16]. The identification of AVM presents differential diagnosis criteria for PWS and KTS. Another overgrowth syndrome, diffuse capillary malformation with overgrowth (DCMO), characterized as reticulate CM contiguously involving multiple anatomic regions with proportionate overgrowth, is similar to KTS and PWS and in the early stage, when the detection of deep vascular lesions such as VMs, LMs, or VMs was challenging, which also hinders early clinical diagnosis. Thence, diagnosis is currently moving from a clinical-histological to a genetic framework, which recognizes that DCMO is caused by somatic *GNA11* mutation, PWS harbors *RASA1* mutations, while KTS is caused by somatic *PIK3CA* and *TEK* mutation [17].

According to the congenital clinical manifestations of the patient described in this case, including bilateral tissue hypertrophy and overgrowth of lower limbs, extensive capillary malformations on both lower and upper limbs, chest and back, significantly increased skin temperature of lesions in both lower limbs than that found in the adjacent normal skin and peripheral venous dilatation suggesting potential fast-flow lesions. Thus, the patient was initially diagnosed with Parkes-Weber syndrome. Subsequent MRI and DSA ruled out the presence of low-flow vascular lesions (venous and lymphatic malformation) or fast-flow malformations (AVM and AVF). A genetic study showed a somatic *PIK3CA* (p.Cys378Arg; c.1132 T > C) mutation. Other vascular diseases associated with *PIK3CA* mutation such as KTS and CLOVES were excluded due to lack of clinical phenotypes including low-low vascular lesions, congenital lipomatous overgrowth, and epidermal nevus. We diagnosed the patient with a unique PROS masquerading as PWS phenotypes. We hypothesized that the increased skin temperature was caused by chronic phlebitis secondary to venous insufficiency and could be easily overlooked and misdiagnosed as manifestation secondary to fast-flow vascular lesions in this patient.

Detailed medical history and physical examination, imaging (ultrasound, MRI, angiography) to identify the characteristics of the vascular lesion, and possible skin biopsy as well as molecular diagnosis can be helpful in establishing a detailed “clinical-pathological-molecular” diagnosis for complex vascular anomalies associated with tissue overgrowth. Chemotherapy, such as sirolimus and alpelisib, inhibitors of the PI3K/AKT/mTOR pathway [22–24], has proven effective and safe in a variety of *PIK3CA*-related vascular anomalies, such as venous/lymphatic malformations (VM/LM) and PROS, by offsetting the progression of the malformations [25, 26].

## Conclusion

Herein, we report one unique clinically challenging case with bilaterally segmentally distributed CM on both the lower and upper limbs, chest and back, and hypertrophic lower limbs associated with longitudinal growth. Initially, the patient was thought to have PWS because of the increased skin temperature and peripheral venous dilatation of his lower limbs; however, we diagnosed the patient as PROS later based on molecular analysis with a somatic *PIK3CA* (p.Cys378Arg; c.1132 T > C) mutation. We found that PROS can present with clinical phenotypes similar to PWS, which may lead to misdiagnosis. Further imaging diagnosis and molecular diagnosis can be valuable to propose a differential diagnosis between PROS and PWS, to fully understand the condition and to assess the potential risks, complications and prognosis of the disease. Molecular targeted therapies such as sirolimus and alpelisib, targeted inhibitors of mTOR and PI3K, respectively, will be viable treatment options for these conditions in the early stages of development.

## Data Availability

All supporting data of this article are included in the submitted manuscript.

## Abbreviations

PROS: *PIK3CA*-Related Overgrowth Syndrome
PWS: Parks-Weber syndrome
NIH: National Institute of Health
AVMs: Arteriovenous malformations
AVFs: Arteriovenous fistulas
DSA: Digital subtraction angiography
KTS: Klippel-Trenaunay syndrome
CT: Computed tomographic
DSA: Digital subtraction angiography
NGS: Targeted next-generation sequencing
